# Development of an Online Adaptive Parameter Tuning vSLAM Algorithm for UAVs in GPS-Denied Environments

**DOI:** 10.3390/s22208067

**Published:** 2022-10-21

**Authors:** Chieh-Li Chen, Rong He, Chao-Chung Peng

**Affiliations:** Department of Aeronautics and Astronautics, National Cheng Kung University, Tainan 701, Taiwan

**Keywords:** adaptive tuning, GPS-denied environments, vSLAM, stereo vision, inertial measurement unit (IMU), mahony complementary filter, S-PTAM

## Abstract

In recent years, unmanned aerial vehicles (UAVs) have been applied in many fields owing to their mature flight control technology and easy-to-operate characteristics. No doubt, these UAV-related applications rely heavily on location information provided by the positioning system. Most UAVs nowadays use a global navigation satellite system (GNSS) to obtain location information. However, this outside-in 3rd party positioning system is particularly susceptible to environmental interference and cannot be used in indoor environments, which limits the application diversity of UAVs. To deal with this problem, in this paper, a stereo-based visual simultaneous localization and mapping technology (vSLAM) is applied. The presented vSLAM algorithm fuses onboard inertial measurement unit (IMU) information to further solve the navigation problem in an unknown environment without the use of a GNSS signal and provides reliable localization information. The overall visual positioning system is based on the stereo parallel tracking and mapping architecture (S-PTAM). However, experiments found that the feature-matching threshold has a significant impact on positioning accuracy. Selection of the threshold is based on the Hamming distance without any physical meaning, which makes the threshold quite difficult to set manually. Therefore, this work develops an online adaptive matching threshold according to the keyframe poses. Experiments show that the developed adaptive matching threshold improves positioning accuracy. Since the attitude calculation of the IMU is carried out based on the Mahony complementary filter, the difference between the measured acceleration and the gravity is used as the metric to online tune the gain value dynamically, which can improve the accuracy of attitude estimation under aggressive motions. Moreover, a static state detection algorithm based on the moving window method and measured acceleration is proposed as well to accurately calculate the conversion mechanism between the vSLAM system and the IMU information; this initialization mechanism can help IMU provide a better initial guess for the bundle adjustment algorithm (BA) in the tracking thread. Finally, a performance evaluation of the proposed algorithm is conducted by the popular EuRoC dataset. All the experimental results show that the developed online adaptive parameter tuning algorithm can effectively improve the vSLAM accuracy and robustness.

## 1. Introduction

Due to high flexibility and excellent maneuverability, UAVs have become one of the most popular aerial vehicle platforms over the past few years. Moreover, the outdoor applications of UAVs are well known to the public, while this paper focuses on visual positioning in GPS-denied environments. Therefore, several indoor applications of UAVs are particularly illustrated. For example, UAVs can provide appropriate assistance to humans for those high-risk missions such as rescue operations and indoor equipment maintenance in nuclear power plants [1]. Besides, UAVs can also effectively enhance human productivity, such as being used for intelligent warehousing management in large factories or plant care and monitoring in greenhouses [2]. Military indoor inspection, indoor photography and scenes scanning for construction purposes are also common indoor applications for UAVs. In addition, with the booming development of autonomous UAV applications, the underlying technologies that UAVs heavily rely on, such as localization and attitude estimation, have gradually attracted the attention of scholars and have become a research hotspot.

UAVs can obtain location and attitude information through many different types of sensors. Depending on the signal source, the sensors can be classified into outside-in types, such as GNSS, VICON (a motion capture system), laser scanner, ultra-wideband (UWB), Wi-Fi, and so on. However, the information from this type of sensor cannot be acquired without the pre-set external facilities. As a consequence, these outside-in localization methods cannot work properly in unexplored environments. On the contrary, inside-out types, such as onboard cameras, radars, light detection and ranging (LiDAR), etc., can receive information independently without any 3rd equipment. In terms of computational power requirements, even though using inside-out type sensors is more costly to sense the ego-motion of UAVs, they can deal with the autonomous navigation problem in unknown or even dynamic environments compared to outside-in type sensors and have significant advantages in hardware cost considerations as well. Moreover, the inside-out perception strategy offers UAVs the ability to sense the environment, also known as mapping. The mapping capability is essential for real-time obstacle avoidance, trajectory planning, and other related autonomous functions. All in all, it can be inferred that SLAM technology based on the inside-out type sensors will play a pivotal role in the development of autonomous UAVs under GPS-denied environments.

The vSLAM is favored by UAV developers for its lightweight and low-power consumption camera sensors. Moreover, rich color information not only makes the map more valuable but also has excellent potential for removing dynamic obstacles [3,4,5,6]. It is also worth mentioning that the well-known open-source monocular SLAM solutions based on static visual features (indirect methods) are parallel tracking and mapping (PTAM) [7] and ORB-SLAM (1–2) [8,9], while large-scale direct monocular SLAM (LSD-SLAM) [10], semi-direct visual odometry (SVO) [11] and direct sparse odometry (DSO) [12] are based on direct methods. However, using only a monocular camera for positioning inevitably causes scale ambiguity and further makes it difficult to directly provide valid information. Therefore, in practice, additional sensors are needed to compensate for the scale, as in [13,14], where this problem is addressed by using radar and barometer, respectively. Besides, with the popularity of micro-electro-mechanical-system (MEMS), integration between vision and IMU, also known as visual-inertial odometry (VIO), such as monocular visual-inertial system (VINS-Mono) [15] and robust visual inertial odometry (ROVIO) [16], has received high attention in recent years, and has been applied in many novel fields like augmented reality (AR) and virtual reality (VR). Unlike monocular cameras, RGB-D cameras can directly access scale and depth information through structured light or Time-of-Flight (ToF). The associated famous vSLAM open-source solutions include ElasticFusion [17], dense visual odometry (DVO) [18], RGB-D SLAM v2 [19], and KinectFusion [20]. Although RGB-D cameras have a strong ability to build high-dense maps, their weight, size, and price will bring a considerable burden to UAVs compared to general RGB cameras. The short measurement distance by an RGB-D sensor also restricts the size applicability of the operating environment. Thus, stereo cameras with lightweight, low cost, low-power consumption, and long measurement distance are obviously more suitable for UAV perceptions. [21] proposes the stereo multi-state constraint Kalman filter (S-MSCKF), which fuses stereo vision and IMU in a tightly coupled manner to construct a highly robust and real-time localization system, and also solves the problem of large state dimension in the traditional filter-based vSLAM architecture. Based on the parallel multi-threading architecture proposed in [7], Pire et al. divide the overall system into front-end tracking and back-end optimization threads performed in parallel, thereby improving time cost and positioning accuracy [22].

Although vSLAM is capable of achieving effective exploration in a GPS-denied environment, the associated algorithms involve a considerable number of parameters or thresholds. In general, these parameters are not only fixed after system startup but also mostly lack intuitive physical meaning, making it difficult to have a well-founded manual adjustment. For UAVs with aggressive motions or in complex operating environments, these parameters require instant online updates to keep the system in optimal condition. For the above reasons, the concept of dynamic threshold is introduced into the field of vSLAM, such as dynamically adjusting threshold value [23], which controls the selection of keyframe based on field-of-view (FoV) repetition rate. Meanwhile, according to the results of several experiments, it is found that the selection of feature matching threshold has a dominant effect on positioning accuracy. Therefore, how to effectively select the matching threshold online according to different situations will be one of the key discussion issues in this paper.

In fact, not only the vSLAM system has the requirement of online parameter adjustment, but also the Mahony complementary filter [24] for UAVs attitude estimations. As the UAVs are under aggressive motions, the measured acceleration varies drastically, which will cause biased attitude estimation. The biased estimates not only deteriorate the basic flight stability of UAVs, but also lower the vSLAM accuracy. Therefore, this paper proposes to adjust the $K_{p}$ online in Mahony complementary filter to further improve the attitude estimation accuracy, not only to achieve better flight performance of UAVs, but also to prepare for the information fusion with the vSLAM system. The following summarizes the three major problems that are going to be addressed in this paper:

1. Online adaptive parameter tuning for feature matching threshold, which lacks physical meaning.
2. Online adaptive $K_{p}$ gain adjustment for Mahony complementary filter to resist aggressive motions.
3. Fusion and motion compensation loop design between vSLAM and IMU.

Following the above-mentioned issues, the further proposed online adaptive parameter tuning algorithm and motion compensation loop in this paper has the following three primary contributions.

1. The matching threshold and the $K_{p}$ gain, which are not easy to determine via manual tuning, are adjusted adaptively according to the UAVs’ flight status.
2. The proposed online adaptive parameter tuning algorithm can effectively improve the pose estimation accuracy and can enhance frame per second (FPS) by up to 70% and 29%, respectively, in the EuRoC dataset.
3. The developed motion compensation loop subroutine can effectively utilize IMU information to improve the anti-shading robustness of the original vSLAM performance. Moreover, incorporating the presented online adaptive parameter tuning algorithm can further improve the robustness to a higher level.

## 2. The Framework of the vSLAM System

This paper develops a proposed online adaptive parameter tuning algorithm based on the S-PTAM [25], whose performance is comparable to the state-of-the-art ORB-SLAM2 [9] and has better accuracy than stereo large-scale direct SLAM (S-LSD-SLAM) [26]. The most significant feature of S-PTAM is that the overall system involves two independent threads, i.e., the tracking thread and the mapping thread. The former mainly performs real-time online localization, and the latter is responsible for local map optimization. In order to introduce the framework of the proposed algorithm, it is essential to briefly explain several important concepts in these threads and the settings adopted in this paper.

### 2.1. Coordinate Setup

**Figure 1:**
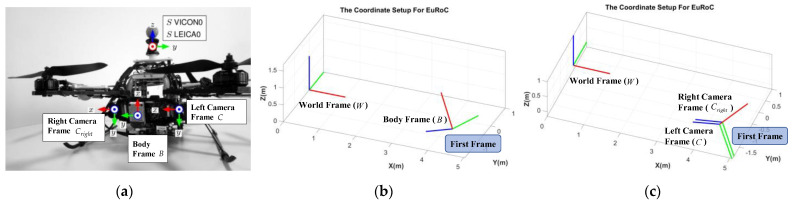
Coordinate setup: (**a**) Illustration of coordinate setup on UAV (modify from [27]); (**b**) Illustration of world frame and body frame; (**c**) Illustration of world frame, right camera frame, and left camera frame.

EuRoC [27] dataset is used for the final algorithm validation. The euRoC dataset not only provides stereo image sequences and IMU measurements but also the relative pose relationships between sensors and the ground truth about 6 degrees of freedom (DoF) pose, which will be used as a reference in this paper. More details about this dataset can be accessed in the “Data Availability Statement” section at the end of this paper. In order to facilitate the comparison of results, this paper refers to its coordinate settings. The whole coordinates system contains a left camera frame (C), right camera frame ($C_{right}$), body frame (B), world frame (W), and navigation frame (S). Both camera frames take the front shooting direction as the *z*-axis, while the right and bottom of the shooting direction as the *x*-axis and *y*-axis, respectively. The optical center of the camera is taken as the origin. The body frame is the same as the *z*-axis parallel to the front shooting direction but different from the camera frames; the upper and right sides of the shooting direction are the *x*-axis and the *y*-axis. The world frame whose *z*-axis is parallel to gravity is defined by instruments that provide ground truth poses, such as the Vicon tracking system or Leica MS50. In order to show the definition of all frames more clearly, Figure 1 is provided to help to understand.

**Figure 2:**
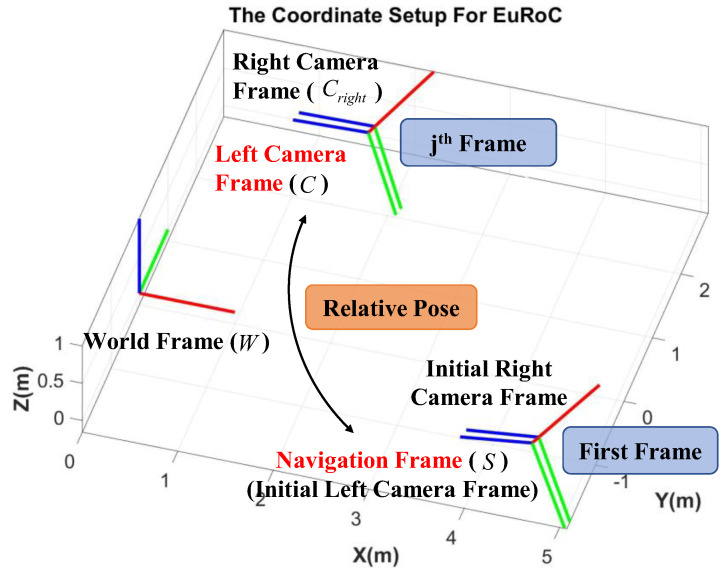
Illustration of navigation frame.

It must be noted that the vSLAM system does not know any information about the world frame during the positioning process, which means that the world frame cannot be used as the reference frame to describe the pose, so it is necessary to introduce the navigation frame. In this paper, the first left camera frame, after activating the vSLAM system, is defined as the navigation frame, and all subsequent positioning information is described as the left camera frame with respect to the navigation frame, as shown in Figure 2.

### 2.2. Keyframe Selection

**Figure 3:**
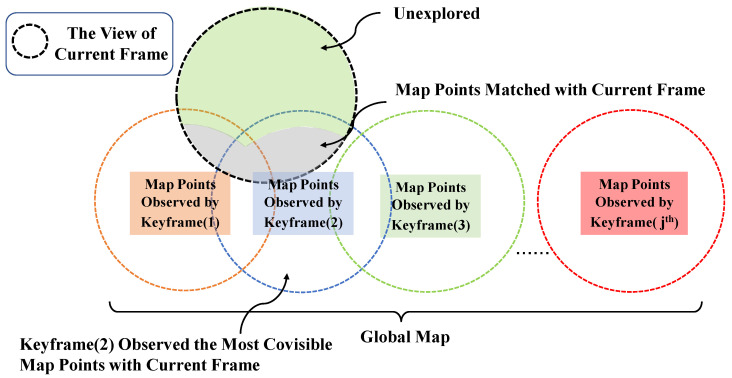
Keyframe selection criteria.

The current tracking frame is treated as a keyframe as long as it meets the keyframe selection criteria. It is worth mentioning that the core strategy of keyframe selection is to check whether the whole map can provide enough feature points for the current tracking frame to match. S-PTAM in [25] selects the keyframe that has the most covisible points with the current tracking frame and then takes the number of map points observed by this keyframe as the reference. When the number of map points matched with the current tracking frame is less than half of the reference or less than twenty, the tracking frame is considered a keyframe. In short, the keyframe observes more places that have not been explored before, as shown in Figure 3.

### 2.3. Tracking Thread

**Figure 4:**
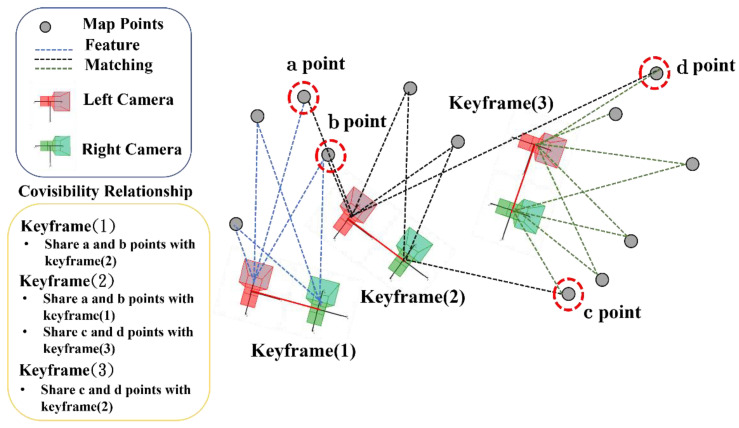
Illustration of covisibility relationship.

S-PTAM [25] will use the feature matching information between keyframes and map points to maintain a covisibility relationship to manage the situation that each map point observed by multiple keyframes, as shown in Figure 4.

**Figure 5:**
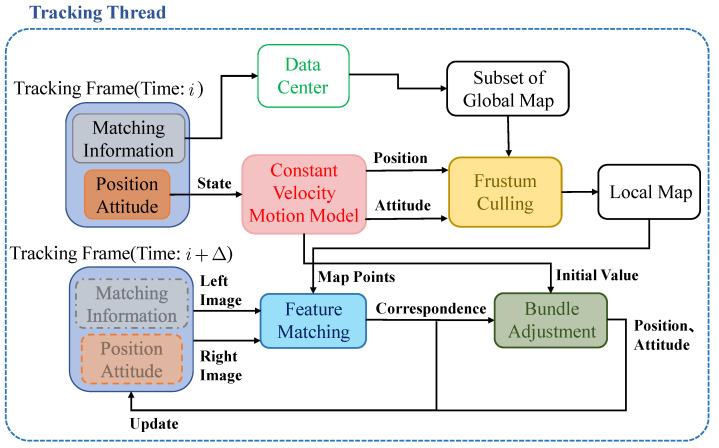
Flowchart of tracking thread.

By searching for keyframes that have a covisibility relationship with the previous tracking frame, the map points that may be observed at that moment can be quickly predicted from the global map, namely the submap. Then, the initial guess of the left camera pose can be predicted by the constant velocity motion model, which can combine with frustum culling to further remove the map points that cannot be seen in the submap, and further obtain the local map. Thereafter, matching features between the stereo image at the moment and the local map are conducted. Finally, according to the matching result, BA is applied to obtain the optimal pose, which takes the output of the constant velocity motion model as the initial guess. The overall tracking thread is to repeat the above steps continuously. The associated detailed flowchart is summarized in Figure 5.

There are two noteworthy points for the aforementioned algorithm flow chart:

- The output of the constant velocity motion model may be a weak initial guess, especially when UAVs are in aggressive motions such as sharp turnings or lost image information.
- The optimization of the BA is highly dependent on the accuracy of feature matching.

In other words, the initial guess and the outliers of the matching pair must be handled carefully during the development process. For the former, this paper will obtain a more reliable value by using IMU information, which will be explained clearly in Section 4 and Section 5. First of all, let’s introduce feature matching and BA in the following section.

### 2.4. Feature Extraction and Matching

**Figure 6:**
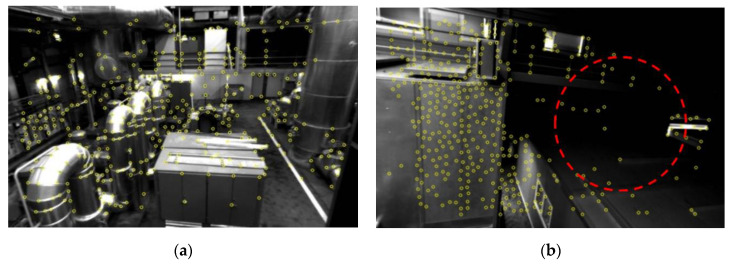
The implementation of the Shi-Tomasi corner detector: (**a**) Detection results in a well-illuminated environment; (**b**) Detection results in a low-illuminated environment.

Before feature matching, feature extraction is performed on the stereo images to find the 2D static feature points, which consist of two parts, key points and descriptors. It is worth mentioning that the key points cannot be matched directly, while the further extracted descriptors allow the key points have the ability to describe the surrounding areas, which makes it possible to perform feature matching. In this paper, the Shi-Tomasi corner detection algorithm [28] proposed by Jianbo and Tomasi is used for the detection of the key point, while binary robust independent elementary features (BRIEF) [29] is used for the descriptors extraction. Figure 6. shows the key point detection results for the EuRoC dataset. Examining Figure 6b, it is obvious that in the low-illuminated area, fewer key points will be detected, which may further lead to positioning divergence or drift.

**Figure 7:**
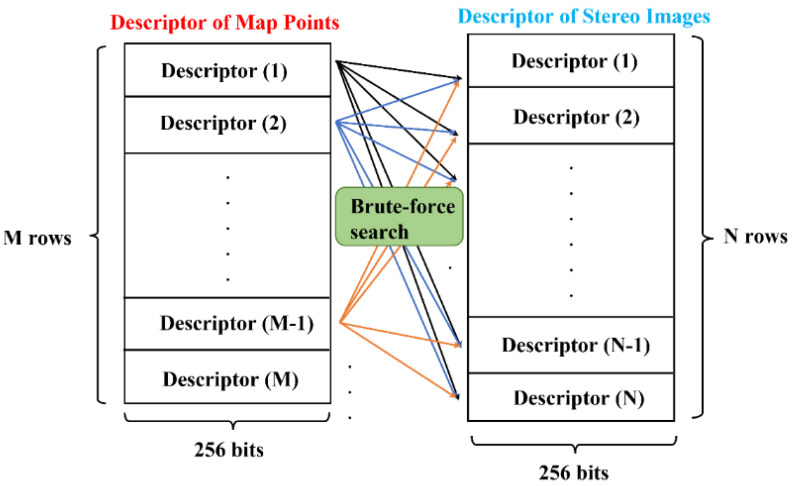
Brute-force feature matching.

**Figure 8:**
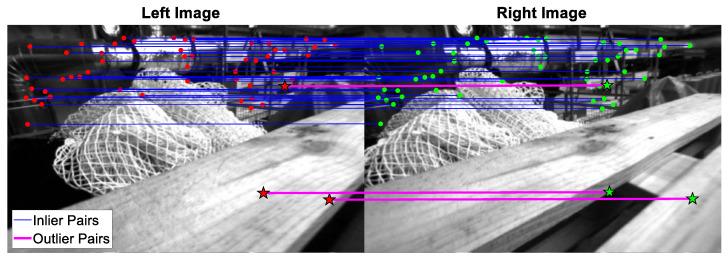
The result of feature matching pairs in the EuRoC dataset. (The left and right image represent the images from the left and right camera of the stereo camera, respectively.)

The BRIEF descriptor is a 256-bit binary vector, therefore, the Hamming distance is adopted to evaluate the feature similarity. For example, if there are two binary vectors, such as 10001 and 01011, with three different bits in the same position between them, then the Hamming distance is defined as three. As a result, the larger the Hamming distance, the lower the similarity of the two vectors. Following the above description, in this paper, each 3D map point on the local map is checked against all the feature points on the current stereo images by the brute-force feature matching algorithm, further matching those with the minimum Hamming distance and below the matching threshold. The illustration of the overall matching algorithm is shown in Figure 7, while Figure 8 shows the result of feature matching in the EuRoC dataset. At the same time, since stereo rectification is performed in advance, the ideal matching pairs can be guaranteed to have close v values, which can be used as a physical constraint to preliminarily filter out the extreme outliers. However, as illustrated in Figure 8, although this strong matching constraint can remove most of the outliers, certain mismatching pairs remain inevitable, as shown by the pink lines in Figure 8. Therefore, the BA algorithm, which is susceptible to mismatching, must introduce an additional mechanism to maintain the pose estimation accuracy. The related details will be presented in the next section.

### 2.5. Bundle Adjustment

Before constructing the BA cost function, the camera model must be established first. The pinhole model is often used to describe the conversion mechanism between the camera frame and the pixel frame. The projection can be expressed as:

(1) $$\left[\begin{array}{c} u\\ v \end{array}\right]=\frac{1}{s}\left[\begin{array}{ccc} f_{x} & 0 & c_{u}\\ 0 & f_{y} & c_{v} \end{array}\right]{\left(\left[\begin{array}{cc} {}_{R}^{C}R & t\\ 0 & 1 \end{array}\right]\left[\begin{array}{c} p^{R}\\ 1 \end{array}\right]\right)}_{1:3}≜h\left({}_{R}^{C}T,p^{R}\right),$$

where u, v are pixel coordinate values, while $f_{x}$, $f_{y}$ are focal lengths (in pixels) in the u and v directions, respectively. $c_{u}$ and $c_{v}$ are the corresponding principal points. ${}_{R}^{C}R$ and t are the rotation matrix and translation vector, respectively, for the reference frame (R) with respect to the camera frame (C), $p^{R}$ is the map point position represented in the reference frame, s is the scale factor, the subscript ${\left(\cdot \right)}_{1:3}$ means to take the first three values of the vector, and ${}_{R}^{C}T$ is a transform matrix that can be written as:

(2) $${}_{R}^{C}T=\left[\begin{array}{cc} {}_{R}^{C}R & t\\ 0 & 1 \end{array}\right]\in \mathrm{SE}\left(3\right),$$

In essence, the BA algorithm is to solve for the optimal camera poses and map point positions by minimizing the re-projection error and its cost function can be expressed as Equation (3).

(3) argminTSCj,est pi,estSJ=∑k=1mukvk−hTSCj,est,pi,estS2

where TSCj,est is the transform matrix for the navigation frame with respect to the $j^{th}$ camera frame, $p_{i,est}^{S}$ is the map point position represented in the navigation frame, and $u_{k}$, $v_{k}$ are the pixel value on the $j^{th}$ camera which match to the map point $p_{i,est}^{S}$. And re-projection error can be defined as:

(4) ek=ukvk−hTSCj,est,pi,estS,

where $e_{k}\in {\mathbb{R}}^{2\times 1}$. Substitute Equation (4) into Equation (3) and convert it into a vector form.

(5) $$J=e_{re}^{T}e_{re},$$

where $e_{re}$ can be written as:

(6) $$e_{re}=\left[\begin{array}{c} e_{1}\\ \vdots \\ e_{m} \end{array}\right]\in {\mathbb{R}}^{2m\times 1},$$

After defining the cost function, the derivatives of the state, i.e., camera poses TSCj,est and map points, $p_{i,est}^{S}$ must be determined in order to construct the Jacobian matrix of $e_{re}$ that will be used in the optimization procedure. However, it is worth noting that the rotating matrix itself has six constraints, that is, the L2-norm of each column and row must equal 1. In other words, it must guarantee that these constraints always hold during each iteration of the optimization, which will not only make the process difficult to perform but also increase the time spent. Fortunately, by introducing Lie algebra [30], this problem can be solved perfectly.

Firstly, define a small pose perturbation as shown in Equation (7).

(7) $$\Delta T_{j,est}=\left[\begin{array}{cc} \Delta R & \Delta t\\ 0 & 1 \end{array}\right]\in \mathrm{SE}\left(3\right),$$

Equation (7) can be written in the form of Lie algebra by logarithmic mapping, shown as follows.

(8) $$\xi_{j,est}=\left[\begin{array}{c} \rho \\ \varphi \end{array}\right]={\left(\ln\left(\Delta T_{j,est}\right)\right)}^{\vee_{SE3}}\in \mathrm{se}\left(3\right),$$

where $\rho \in {\mathbb{R}}^{3\times 1}$, $\varphi \in {\mathbb{R}}^{3\times 1}$, and ${\left(\cdot \right)}^{\vee_{SE}}$ is defined as:

(9) $${\left[\begin{array}{cccc} 0 & -a_{6} & a_{5} & a_{1}\\ a_{6} & 0 & -a_{4} & a_{2}\\ -a_{5} & a_{4} & 0 & a_{3}\\ 0 & 0 & 0 & 0 \end{array}\right]}^{\vee_{SE3}}=\left[\begin{array}{c} a_{1}\\ a_{2}\\ a_{3}\\ a_{4}\\ a_{5}\\ a_{6} \end{array}\right],$$

Alternatively, Equation (8) can be written as Equation (10) by exponential mapping.

(10) $$\Delta T_{j,est}=\exp\left(\xi_{j,est}^{\wedge_{SE3}}\right),$$

where ${\left(\cdot \right)}^{\wedge_{SE3}}$ is the inverse operation of Equation (9) and can be defined as:

(11) $${\left[\begin{array}{c} a_{1}\\ a_{2}\\ a_{3}\\ a_{4}\\ a_{5}\\ a_{6} \end{array}\right]}^{\wedge_{SE3}}=\left[\begin{array}{cccc} 0 & -a_{6} & a_{5} & a_{1}\\ a_{6} & 0 & -a_{4} & a_{2}\\ -a_{5} & a_{4} & 0 & a_{3}\\ 0 & 0 & 0 & 0 \end{array}\right],\;$$

An additional similar marker ${\left(\cdot \right)}^{\wedge_{SO3}}$ must be introduced, whose definition is shown in Equation (12), and both ${\left(\cdot \right)}^{\wedge_{SE3}}$ ${\left(\cdot \right)}^{\wedge_{SO3}}$ will be used in the subsequent derivation.

(12) $${\left[\begin{array}{c} a_{1}\\ a_{2}\\ a_{3} \end{array}\right]}^{\wedge_{SO3}}=\left[\begin{array}{ccc} 0 & -a_{3} & a_{2}\\ a_{3} & 0 & -a_{1}\\ -a_{2} & a_{1} & 0 \end{array}\right],$$

Then, the derivative of the re-projection error for small pose perturbation (represented as the Lie algebra) can be obtained by the chain rule.

(13) $$\frac{\partial e_{k}}{\partial \xi_{j,est}}=\frac{\partial e_{k}}{\partial p_{i,est}^{C}}\frac{\partial p_{i,est}^{C}}{\partial \xi_{j,est}},$$

where $p_{i,est}^{C}$ is defined as:

(14) pi,estC=TSCj,estpi,estS11:3=RSCj,estpi,estS+tj,est,

Therefore, according to Equations (1) and (14), the first term $\partial e_{k}/\partial p_{i,est}^{C}$ in Equation (13) can be derived as follows.

(15) $$\frac{\partial e_{k}}{\partial p_{i,est}^{C}}=\frac{\partial \left(\left[\begin{array}{c} u_{k}\\ v_{k} \end{array}\right]-h\left(T_{j,est},p_{i,est}^{S}\right)\right)}{\partial p_{i,est}^{C}}=-\left[\begin{array}{ccc} \frac{f_{x}}{p_{i,est,z}^{C}} & 0 & \frac{-f_{x}\cdot p_{i,est,x}^{C}}{{\left(p_{i,est,z}^{C}\right)}^{2}}\\ 0 & \frac{f_{y}}{p_{i,est,z}^{C}} & \frac{-f_{y}\cdot p_{i,est,y}^{C}}{{\left(p_{i,est,z}^{C}\right)}^{2}} \end{array}\right],$$

where $p_{i,est}^{C}={\left[\begin{array}{ccc} p_{i,est,x}^{C} & p_{i,est,y}^{C} & p_{i,est,z}^{C} \end{array}\right]}^{T}$, and the scale factor s is equal to $p_{i,est,z}^{C}$. Next, the left perturbation model will be introduced in order to obtain the second term $\partial p_{i,est}^{C}/\partial \xi_{j,est}$ in Equation (13), which is the derivative of Lie algebra.

(16) ∂pi,estC∂ξj,est=∂(ΔTj,estTSCj,est[pi,estS1])1:3∂[ρTφT]T=∂(exp(ξ∧SE3)TSCj,est[pi,estS1])1:3∂[ρTφT]T≈∂((I+ξ∧SE3)[RSCj,esttj,est01][[pi,estS1]])1:3∂[ρTφT]T=∂([φ∧SO3ρ00][RSCj,estpi,estS+tj,est1])1:3∂[ρTφT]T=∂(φ∧SO3(RSCj,estpi,estS+tj,est)+ρ)∂[ρTφT]T=∂(−(RSCj,estpi,estS+tj,est)∧SO3φ+ρ)∂[ρTφT]T=[I−(pi,estC)∧SO3]

The derivative of the re-projection error with respect to the pose is obtained by substituting Equations (15) and (16) into Equation (13), which yields

(17) $$\frac{\partial e_{k}}{\partial \xi_{j,est}}=-\left[\begin{array}{ccc} \frac{f_{x}}{p_{i,est,z}^{C}} & 0 & \frac{-f_{x}\cdot p_{i,est,x}^{C}}{{\left(p_{i,est,z}^{C}\right)}^{2}}\\ 0 & \frac{f_{y}}{p_{i,est,z}^{C}} & \frac{-f_{y}\cdot p_{i,est,y}^{C}}{{\left(p_{i,est,z}^{C}\right)}^{2}} \end{array}\right]\left[\begin{array}{cc} I & -{\left(p_{i,est}^{C}\right)}^{\wedge_{SO3}} \end{array}\right]\in {\mathbb{R}}^{2\times 6},$$

The derivative of the re-projection error with respect to the map point can be obtained by using the chain rule again, which gives

(18) $$\frac{\partial e_{k}}{\partial p_{i,est}^{S}}=\frac{\partial e_{k}}{\partial p_{i,est}^{C}}\frac{\partial p_{i,est}^{C}}{\partial p_{i,est}^{S}},$$

It is worth noting that the first term $\partial e_{k}/\partial p_{i,est}^{C}$ of Equation (18) has been derived from Equation (15). Therefore, only the second term $\partial p_{i,est}^{C}/\partial p_{i,est}^{S}$ needs to be derived. This result can be obtained directly from Equation (14), as shown in the following.

(19) ∂pi,estC∂pi,estS=∂RSCj,est⋅pi,estS+tj,estpi,estS=RSCj,est,

Substituting Equations (15) and (19) into Equation (18) yields

(20) ∂ek∂pi,estS=−fxpi,est,zC0−fx⋅pi,est,xCpi,est,zC20fypi,est,zC−fy⋅pi,est,yCpi,est,zC2RSCj,est∈ℝ2×3,

According to Equations (17) and (20), the Jacobian matrix of $e_{re}$ can be constructed by

(21) $$J_{e_{re}}=\left[\begin{array}{cc} \frac{\partial e_{1}}{\partial p_{1,est}^{S}}\cdots \frac{\partial e_{1}}{\partial p_{i,est}^{S}} & \frac{\partial e_{1}}{\partial \xi_{1,est}}\cdots \frac{\partial e_{1}}{\partial \xi_{j,est}}\\ \vdots \;\ddots \;\vdots & \vdots \;\ddots \;\vdots \\ \frac{\partial e_{m}}{\partial p_{1,est}^{S}}\cdots \frac{\partial e_{m}}{\partial p_{i,est}^{S}} & \frac{\partial e_{m}}{\partial \xi_{1,est}}\cdots \frac{\partial e_{1}}{\partial \xi_{j,est}} \end{array}\right]\in {\mathbb{R}}^{\left(2m\right)\times \left(3i+6j\right)},$$

Based on the Jacobian matrix Equation (21), the increment of state can be solved by applying Levenberg–Marquardt algorithm (LM) to further obtain the optimal camera poses and map point positions. That is

(22) $$\Delta x=-{\left(J_{e_{re}}^{T}J_{e_{re}}+\lambda I\right)}^{-1}\left(J_{e_{re}}^{T}e_{re}\right),$$

where Δx is defined as:

(23) $$\Delta x={\left[\begin{array}{cc} {\left(\Delta p_{1}^{S}\right)}^{T}\cdots {\left(\Delta p_{i}^{S}\right)}^{T} & {\left(\xi_{1}\right)}^{T}\cdots {\left(\xi_{j}\right)}^{T} \end{array}\right]}^{T},$$

In each optimization iteration, the state will be continuously updated according to Equation (24) until the increment Δx is small enough to stop. Moreover, the termination condition applied in this paper is that the root means square of Δx is less than $10^{-9}$.

(24) pi′,estS=pi′,estS+Δpi′S,i′∈1,i TSCj′,est=expξj′^SE3TSCj′,est,j′∈1,j

However, it is worth noting that BA is sensitive to outliers, namely feature mismatching. To solve this issue, some strategies for filtering outliers must be designed. In this paper, the Huber loss will be used to deal with this problem.

Different from the square error expressed in Equation (3), the cost function is now modified as follows by considering the Huber loss.

(25) J=∑k=1mLukvk−hTSCj,est,pi,estS,

where $L\left(\cdot \right)$ is the Huber function and is defined as:

(26) $$L\left(e\right)=\sum_{i=1}^{n}{\bar{e}}_{i}\;;\;\left\{\begin{array}{l} {\bar{e}}_{i}=\frac{1}{2}e_{i}{}^{2}\;\left|e_{i}\right|\leq \delta \\ {\bar{e}}_{i}=\delta \left|e_{i}\right|-\frac{1}{2}\delta^{2}\;\left|e_{i}\right|>\delta \end{array}\right.,$$

where $e\in {\mathbb{R}}^{n\times 1}$, $e_{i}$ represents the $i^{th}$ element of the vector e and δ is the Huber threshold which is set to 5.991 in this paper and must be set manually in advance. It is evident that when the error is greater than the threshold δ, the error will show a linear growth instead of the original squared increment, which can effectively eliminate the large error caused by the outliers.

A detailed derivation and explanation about how to realize the outliers suppression into the state increment calculation shown in Equation (22) are given in the following. With the use of the Huber function (26), let us redefine the problem as searching for a state increment Δx based on a fixed state $x_{fix}$ that can make the modified cost function as small as possible. That is

(27) $$J=L\left(e_{re}\left(x_{fix},\Delta x\right)\right),$$

By using the Taylor expansion, the first-order approximation can be obtained

(28) $$J\approx L\left({\bar{e}}_{re}\right),$$

where ${\bar{e}}_{re}$ is defined as:

(29) $${\bar{e}}_{re}=e_{re}\left(x_{fix}\right)+J_{e_{re}}\Delta x,$$

Using the chain rule, the derivative of the cost function with respect to the state increment can be expressed by

(30) $$\begin{array}{l} \frac{\partial L\left({\bar{e}}_{re}\right)}{\partial \Delta x}={\left(\frac{\partial \left({\bar{e}}_{re}\right)}{\partial \Delta x}\right)}^{T}\mathrm{diag}\left({\bar{e}}_{re}\right){\left(\mathrm{diag}\left({\bar{e}}_{re}\right)\right)}^{-1}\frac{\partial L\left({\bar{e}}_{re}\right)}{\partial \left({\bar{e}}_{re}\right)}\\ =J_{e_{re}}^{T}\mathrm{diag}\left({\bar{e}}_{re}\right)b=J_{e_{re}}^{T}B{\bar{e}}_{re} \end{array}$$

where b and B are defined by

(31) $$b={\left(\mathrm{diag}\left({\bar{e}}_{re}\right)\right)}^{-1}\frac{\partial L\left({\bar{e}}_{re}\right)}{\partial \left({\bar{e}}_{re}\right)},$$

and

(32) $$B=\mathrm{diag}\left(b\right),$$

respectively.

According to Equations (26) and (31), the detailed configuration of the diagonal matrix B can be written as:

(33) $$B=\left[\begin{array}{cccc} \psi \left({\bar{e}}_{re,1}\right) & 0 & \cdots & 0\\ 0 & \psi \left({\bar{e}}_{re,2}\right) & \cdots & 0\\ \vdots & \vdots & \ddots & \vdots \\ 0 & 0 & \cdots & \psi \left({\bar{e}}_{re,2m}\right) \end{array}\right],$$

where ${\bar{e}}_{re,1}$, ${\bar{e}}_{re,2}$ and ${\bar{e}}_{re,2m}$ are the 1st, 2nd and 2*mth* element of the vector ${\bar{e}}_{re}$, respectively. The $\psi \left(\cdot \right)$ is defined as:

(34) $$\psi \left(e\right)=\left\{\begin{array}{l} 1\;\left|e\right|\leq \delta \\ \frac{\delta }{\left|e\right|}\;\left|e\right|>\delta \end{array}\right.,$$

Substituting Equation (29) into Equation (30) yields

(35) $$\frac{\partial L\left({\bar{e}}_{re}\right)}{\partial \Delta x}=J_{e_{re}}^{T}B{\bar{e}}_{re}=J_{e_{re}}^{T}B\left(e_{re}\left(x_{fix}\right)+J_{e_{re}}\Delta x\right),$$

If Equation (35) is zero, one has

(36) $$\begin{array}{l} J_{e_{re}}^{T}B\left(e_{re}\left(x_{fix}\right)+J_{e_{re}}\Delta x\right)=0\\ \Rightarrow \Delta x=-{\left(J_{e_{re}}^{T}BJ_{e_{re}}\right)}^{-1}J_{e_{re}}^{T}Be_{re}\left(x_{fix}\right) \end{array}$$

Compared with Equation (22), obviously, the cost function with Huber loss is almost equivalent to iteratively reweighted least squares (IRLS). Therefore, based on Equations (36) and (24), the BA algorithm with outlier rejection can be carried out to obtain the optimal camera poses and map point positions.

Following the above description, not only the sensitivity of the BA with respect to the initial value but also the resistance against outliers will be tested. More specifically, the ground truth pose of the camera (TSCgt) is multiplied by a perturbation as the initial value, which is shown in Equation (37).

**Figure 9:**
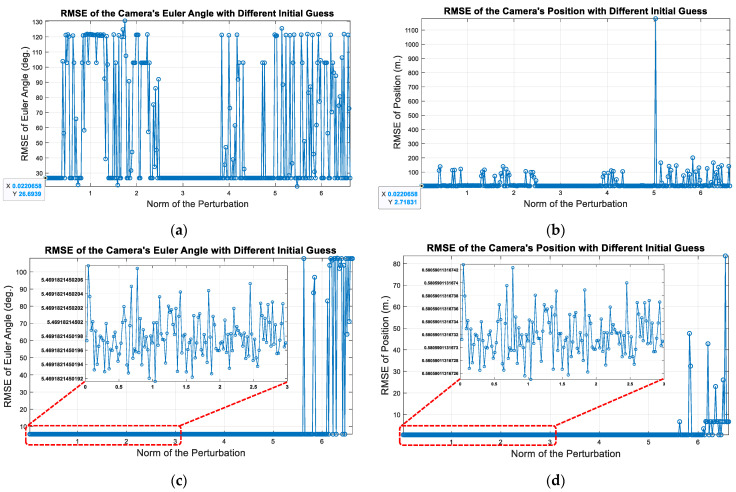
Optimization results of motion-only BA according to different initial guesses: (**a**) RMSE of Euler angle (without using Huber); (**b**) RMSE of position (without using Huber); (**c**) RMSE of Euler angle (with using Huber); (**d**) RMSE of position (with using Huber).

(37) TSCinitial guess=TSCgtexpζ∧SE3,

where $\zeta \in se\left(3\right)$. Finally, we plot the root mean square error (RMSE) of the estimated camera pose according to different initial guesses TSCinitial guess, which are represented as different L2-norm values ζ. At the same time, it must be emphasized that the BA used in the tracking thread does not optimize the numerous map points, but only the current camera poses in order to improve real-time performance, also known as motion-only BA [8]. Therefore, the following tests (with outliers) are all based on this type of BA. The first row of Figure 9 shows the results without Huber, while the second row presents those with Huber.

Apparently, with the aid of the Huber loss, the performance of BA is much more accurate, which means that the Huber loss can effectively resist the impact of outliers. Meanwhile, the sensitivity against the initial value perturbation is also much lower. At the right side of the horizontal axis of each graph in Figure 9, it also illustrates that if the initial guesses are quite poor, the estimated state will still diverge even using the Huber loss. Fortunately, the image frame rate is mostly between 20 and 30. The UAV’s pose state usually does not change much in such a short period of time (about 0.05 s). Therefore, a terrible initial guess is less likely to be created under normal circumstances.

## 3. Online Adaptive Matching Threshold Tuning for vSLAM System

### 3.1. Accuracy Analysis under Different Matching Thresholds

After introducing the problems faced by BA, let’s move on to another issue. Feature matching plays a quite important role in the feature-based vSLAM system, and the matching result will directly affect many critical procedures, such as keyframe selection, BA, triangulation used in mapping, and so on. Undoubtedly, all of the above will directly or indirectly impact the final vSLAM accuracy. Meanwhile, as mentioned in Section 2.4, feature matching relies heavily on the matching threshold, which is a Hamming distance with no physical meaning and must be set before starting the vSLAM system. Based on the above description, a correlation between the matching threshold and the positioning accuracy definitely exists. As a result, a guide regarding the selection of the threshold should be provided.

Take the MH_01_easy series as an example, and observe the positioning accuracy with matching thresholds 10, 15 and 20, respectively. As listed in Table 1, the absolute trajectory error (ATE) and relative pose error (RPE) [31] are adopted as the benchmark to evaluate the accuracy.

According to Table 1, it can be seen that the RPE and ATE are not the smallest for the most severe matching threshold, 10. The reason is that when the matching threshold is very small, it drastically reduces the number of correct match pairs and thus loses the constraint for state optimization. However, the increase in the matching threshold does not necessarily guarantee an increase in localization accuracy. Table 1 clearly reveals that a robust vSLAM should be able to adjust the associated matching threshold automatically. The online automatic threshold scheduling not only improves the overall localization accuracy but prevents tedious manual adjustment as well. In the following section, an online adaptive matching threshold tuning algorithm is proposed.

### 3.2. Online Adaptive Matching Threshold Tuning Algorithm

**Figure 10:**
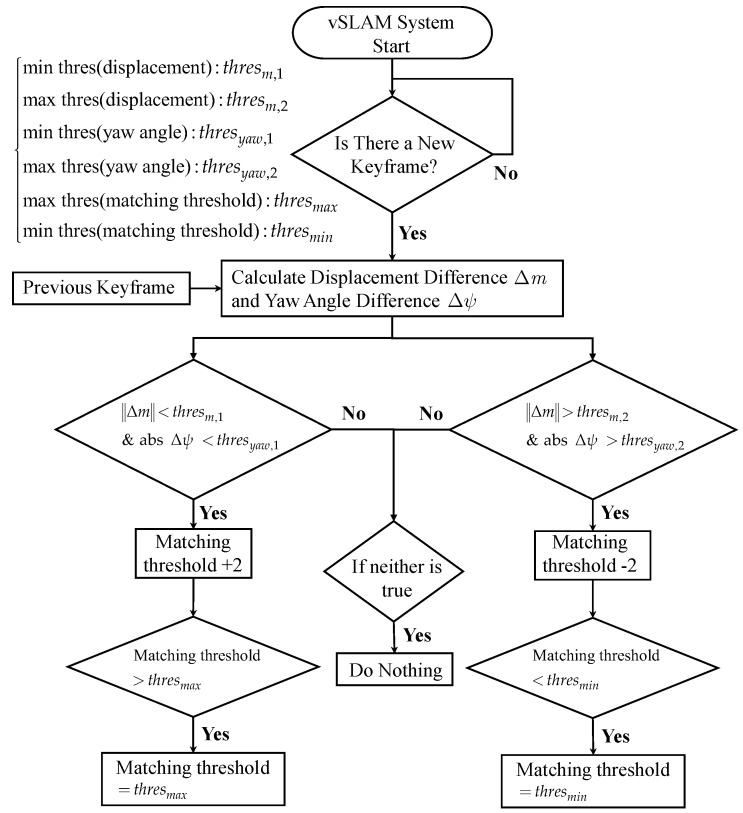
Flowchart of online adaptive matching threshold tuning algorithm.

This research tends to use more physically meaningful displacement and yaw angle differences between keyframes as indicators to adjust the matching threshold. As mentioned in Section 2.2, the overlap rate between the keyframe’s FoV and the current global map is relatively low. For example, when the displacement and yaw angle difference between two adjacent keyframes are less than certain thresholds defined as $thres_{m,1}$ and $thres_{yaw,1}$, it represents a contradiction with the definition of a keyframe. Therefore, it can be inferred that the threshold of the Hamming distance is too severe, and then increases the value immediately. In the opposite case, the matching threshold will be reduced. Because the initial stereo image is the most stable, the initial matching threshold is set to 10, and an upper bound $thres_{max}$ and lower bound $thres_{min}$ are set to limit the matching threshold range. The overall process is summarized in Figure 10, and the parameter settings used in this paper are listed in Table 2.

## 4. Online Adaptive Parameter Tuning for Mahony Complementary Filter

### 4.1. Mahony Complementary Filter

IMUs is an indispensable component for UAVs, and most flight control algorithms rely heavily on them for attitude estimation. In other words, IMU information is basically available on UAVs. Besides, it is expected that the positioning accuracy or robustness can be improved by fusing IMU and vSLAM information. Therefore, this paper not only uses vSLAM to obtain positioning information but also uses Mahony complementary filter to calculate the UAV’s attitude from IMU measurements. First, a brief introduction to the Mahony complementary filter will be given below.

The first step to carrying out the Mahony complementary filter algorithm is to integrate the gyroscope measurements or angular velocity, which can be expressed as

(38) qG¯Bk=normalizeqG¯Bk−1+0.5ΔkΩωk−1,mesqG¯Bk−1,

where $\bar{G}$ represents the gravity frame, whose *z*-axis is always parallel to the gravity. qG¯Bk is the normalized quaternion, which represents the attitude of the gravity frame with respect to the body frame at the moment k. $\omega_{k-1,mes}$ is the angular velocity measurement at the moment k−1 and Δk is the sampling period of the IMU, which is 0.005 s in the EuRoC dataset. $\Omega \left(\cdot \right)$ is defined as:

(39) $$\Omega \left(\omega \right)=\left[\begin{array}{cccc} 0 & -\omega_{x} & -\omega_{y} & -\omega_{z}\\ \omega_{x} & 0 & \omega_{z} & -\omega_{y}\\ \omega_{y} & -\omega_{z} & 0 & \omega_{x}\\ \omega_{z} & \omega_{y} & -\omega_{x} & 0 \end{array}\right]\;;\;\omega =\left[\begin{array}{c} \omega_{x}\\ \omega_{y}\\ \omega_{z} \end{array}\right],$$

The unit gravity vector ${\bar{a}}_{k,est}$ is further estimated by the attitude qG¯Bk derived from Equation (38), as shown in Equation (40).

(40) a˜k,est=qG¯Bk*⊗0001⊗qG¯Bk ; a˜k,est=0a¯k,est,

where ${\bar{a}}_{k,est}$ is the estimated unit gravity vector, the star mark in qG¯Bk* represents the conjugate operation on the quaternion, and ⊗ is defined as:

(41) $$\begin{array}{l} {\left[\begin{array}{cccc} q_{1} & q_{2} & q_{3} & q_{4} \end{array}\right]}^{T}⊗{\left[\begin{array}{cccc} {q^{\prime }}_{1} & {q^{\prime }}_{2} & {q^{\prime }}_{3} & {q^{\prime }}_{4} \end{array}\right]}^{T}\\ =\left[\begin{array}{c} q_{1}{q^{\prime }}_{1}-q_{2}{q^{\prime }}_{2}-q_{3}{q^{\prime }}_{3}-q_{4}{q^{\prime }}_{4}\\ q_{1}{q^{\prime }}_{2}+q_{2}{q^{\prime }}_{1}+q_{3}{q^{\prime }}_{4}-q_{4}{q^{\prime }}_{3}\\ q_{1}{q^{\prime }}_{3}-q_{2}{q^{\prime }}_{4}+q_{3}{q^{\prime }}_{1}+q_{4}{q^{\prime }}_{2}\\ q_{1}{q^{\prime }}_{4}+q_{2}{q^{\prime }}_{3}-q_{3}{q^{\prime }}_{2}+q_{4}{q^{\prime }}_{1} \end{array}\right] \end{array}$$

After obtaining the estimated unit gravity vector, the error $e_{k}$ can be calculated by comparing ${\bar{a}}_{k,est}$ it with the acceleration measurement

(42) $$e_{k}={\bar{a}}_{k,est}\times \frac{a_{k,filter}}{\left\|a_{k,filter}\right\|},$$

where $a_{k,filter}$ represents the acceleration measurement after passing through a low-pass filter (LPF) at moment k. The setting of the LPF in this paper will be described in Section 5.1. Then, a P gain $K_{p}$ is applied for IMU pose estimation correction purpose

(43) $$u_{k}=K_{p}e_{k},$$

The correction effort $u_{k}$ is used to compensate for the angular velocity measurement. As a result, the new attitude estimation at the next moment can be derived by integrating the compensated angular velocity shown as follows

(44) qG¯Bk+1=normalizeqG¯Bk+0.5ΔkΩωk,mes−ukqG¯Bk,

However, there are some problems in this process, which will be analyzed and improved in the following section.

### 4.2. Online Adaptive $K_{p}$ Tuning

According to Equations (42) and (43), the attitude error correction (44) should have a premise. The acceleration measurement is supposed to contain gravity information only to truly reflect the accumulated error of attitude. In other words, the compensation timing of Equation (44) should be restricted. Put simply, when the difference between acceleration measurement and gravity is greater than a threshold $thres_{norm}$, the compensation effort $u_{k}$ should be disabled.

Based on the above description, a confidence level is considered to represent the difference between the acceleration measurement and gravity. For example, the smaller the difference between the two, the less the UAV’s acceleration there is in acceleration measurement, which can reflect the accumulated error more truly. Based on this motivation, this paper further proposes an adaptive $K_{p}$, applied in Equation (43), in compliance with different motions in order to improve the estimation accuracy.

The following presents several adjustment strategies (including control and experimental group) to analyze the accuracy improvement.

- Pure Integration (control group):

The attitude is directly obtained by integrating the angular velocity according to Equation (38).

- Arctan Method (control group):

Directly obtain the Euler angle directly through Equation (45).

(45) $$\begin{array}{l} \phi =\mathrm{atan}2\left(a_{k,y,filter},a_{k,z,filter}\right)\\ \theta =\mathrm{atan}2\left(-a_{k,x,filter},\sqrt{a_{k,y,filter}^{2}+a_{k,z,filter}^{2}}\right) \end{array}$$

- Pure Mahony (control group):

No matter if the difference between acceleration measurement and gravity is smaller than the threshold $thres_{norm}$, the angular velocity is always compensated according to Equation (44).

- Conditional Method (experimental group):

If the difference between acceleration measurement and gravity is greater than the threshold $thres_{norm}$, $u_{k}$ will be set to zero.

- Adaptive Method Version. 1 (experimental group):

Based on the Conditional Method, $K_{p}$ in Equation (43) is further adjusted according to Equation (46).

(46) $$u_{k}=K_{p}\cdot \exp\left(-\frac{\mathrm{abs}\left(\left\|a_{k,filter}\right\|-g\right)}{thres_{norm}}\right)e_{k},$$

where g is gravity acceleration and is set to 9.82121 in this paper.

- Adaptive Method Version. 2 (experimental group):

Based on the Adaptive Method Version.1, a minor change is made

(47) $$u_{k}=K_{p}\cdot \exp\left(-\frac{\mathrm{abs}\left(\left\|a_{k,filter}\right\|-g\right)}{\kappa \cdot thres_{norm}}\right)e_{k},$$

where κ is an additional parameter to adjust the sensitivity of $K_{p}$ for acceleration change.

- Adaptive Method Version. 3 (experimental group):

Based on the Adaptive Method Version.2, modify Equation (47) as the following adaption law

(48) $$u_{k}=\left(K_{p}+\Delta K_{p}\cdot \exp\left(-\frac{\mathrm{abs}\left(\left\|a_{k,filter}\right\|-g\right)}{\kappa \cdot thres_{norm}}\right)\right)e_{k},$$

**Figure 11:**
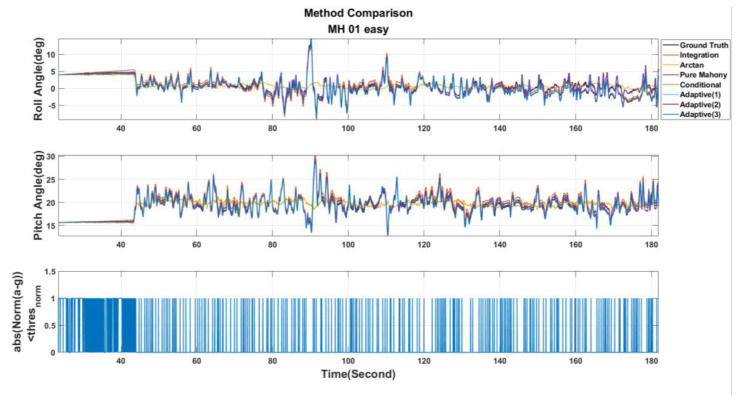
Roll and pitch angle obtained from different adjustment strategies for MH_01_easy series.

**Figure 12:**
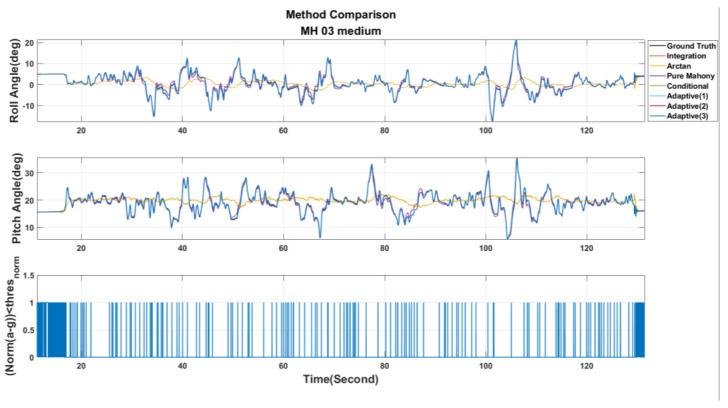
Roll and pitch angles were obtained from different adjustment strategies for the MH_03_medium series.

**Figure 13:**
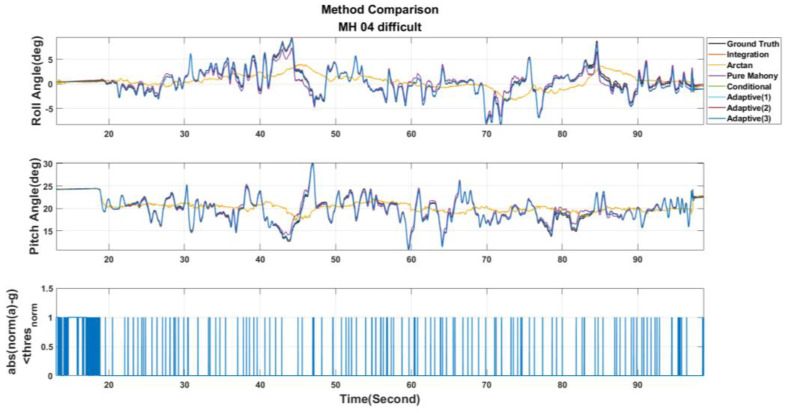
Roll and pitch angles were obtained from different adjustment strategies for the MH_04_difficult series.

Table 3 shows the parameter settings used in this paper. Then, the EuRoC dataset is used to evaluate the above seven adjustment strategies. Besides, the estimated results are shown in Figure 11, Figure 12 and Figure 13. These figures show the roll and pitch angle estimation results for different adjustment strategies and are presented in the first and second rows of each figure, respectively, while the third row represents a flag that indicates whether the difference between measured acceleration and gravity is smaller than the threshold $thres_{norm}$. Finally, the RMSE of the Euler angle is listed in Table 4.

Examining Table 4, it can be observed that Pure Mahony achieved good accuracy performance in the MH_01_easy and MH_02_easy series. The main reasons are further analyzed. According to [27], it can be known that the UAV’s motions in both the series are relatively slow in the whole dataset, which means that the error $e_{k}$ is reliable most of the time, so the Pure Mahony, which always compensates the angular velocity, will have high accuracy in attitude estimation. At the same time, we observe that the Conditional Method has quite higher accuracy than Pure Mahony for aggressive motions series, such as MH_03_medium, MH_04_difficult and MH_05_difficult. These experimental results strongly show that conditional compensation can improve accuracy in aggressive motions definitely in regard to the Adaptive Method Version. 1~3, we can find that the Adaptive Method Version.3 has more stable and accurate results, which sufficiently shows that the online $K_{p}$ adjustment is helpful for the attitude estimation in aggressive motions. Finally, in the MH_04_difficult series, the results show that the Pure Integration achieves a good accuracy performance in the roll angle; this is because the EuRoC dataset uses a higher grade IMU. According to [27], MH_04_difficult has the shortest duration among the five series, so the accumulation error induced by pure integration is not significant. There is no doubt that using pure integration to obtain the attitude will definitely cause estimation drift. Briefly, this paper will adopt the Adaptive Method Version. 3 for the attitude calculation.

## 5. Motion Compensation Loop Design

### 5.1. Static State Detection Algorithm

With the above introduction and analysis of the Mahony complementary filter, it is obvious that the attitude calculated through the IMU is relative to the gravity frame, while the information obtained by vSLAM is relative to the navigation frame. Therefore, information alignment from the IMU gravity frame to the vSLAM navigation frame is essential for pose compensation.

Equation (49) shows the process of converting the IMU information into the same reference frame as vSLAM.

(49) TCSk,imu=TB0STG¯B0TBG¯k,imuTCB,

where TBG¯k,imu is the $k^{th}$ pose information for the body frame relative to the gravity frame calculated by the IMU, TCSk,imu is the converted IMU information, and $B_{0}$ is the first body frame. Besides, according to the definition of the navigation frame, TB0S is the inverse matrix of TCB, which represents the relative pose between the body frame and the left camera frame, and it is worth mentioning that TB0S and TCB are fixed values only related to the hardware setup, and must be derived from the calibration procedure in advance. Fortunately, in the EuRoC dataset, these values have been precisely provided. It is obvious that each conversion will involve TG¯B0. In other words, it must be as accurate as possible in order to avoid negative compensation effects. Meanwhile, when a UAV is stationary, the acceleration measurements which do not involve linear acceleration are much cleaner. Based on the stationary property of the UAV, accelerometer measurements in a static state will be applied to obtain accuracy TG¯B0 before activating the vSLAM system. First, in order to know when the UAV is stationary exactly, this paper further proposes a static state detection algorithm, which will be illustrated in detail below.

However, even in the stationary status, the accelerometer inevitably contains high-frequency noise and thereby causes inaccurate pose estimation. An LPF, as shown in Equation (50), is applied to suppress the high-frequency noise appearing in the acceleration measurements

(50) $$a_{k,filter}=\beta a_{k-1,filter}+\left(1-\beta \right)a_{k,mes},$$

where $a_{k,filter}$ is the $k^{th}$ filtered acceleration measurement, $a_{k,mes}$ is the $k^{th}$ unfiltered acceleration measurement, while β can be defined as:

(51) $$\beta =\frac{1}{\left(1+2\pi f_{c}\Delta k\right)},$$

where $f_{c}$ is the cutoff frequency and is set to be 0.4775 Hz in this paper.

**Figure 14:**
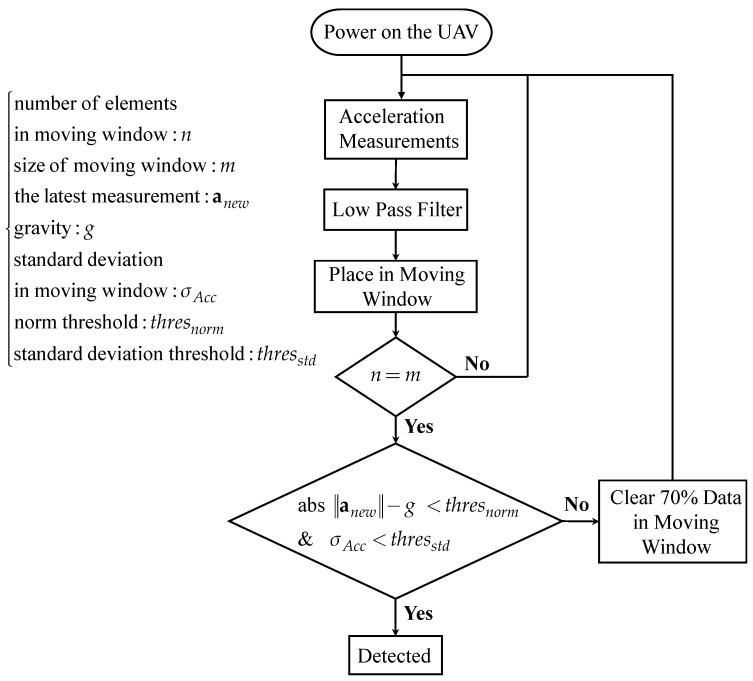
Flowchart of the proposed static state detection algorithm.

**Figure 15:**
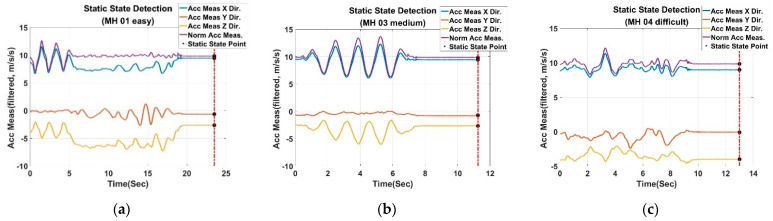
Filtered acceleration measurements and the results of static state detection: (**a**) For MH_01_easy series; (**b**) For MH_03_medium series; (**c**) For MH_04_difficult series.

After filtering the acceleration measurements, they are placed into a moving window (500 pieces in size). When the moving window is full, the judgment procedure will be triggered. If the standard deviation in this moving window is less than $thres_{std}$, which is set to be 0.02, and the difference between the latest measurement and the gravity is less than $thres_{norm}$ as well, the moment is considered to be stationary. However, if the condition is not satisfied, then 70% history data in the moving window will be cleared, and the above action will be repeated again until the static state is detected. The flowchart of the overall detection algorithm is illustrated in Figure 14, while Figure 15 shows the results of the static state detection for the EuRoC dataset. In Figure 15, the filtered 3-axis acceleration and the L2-norm of acceleration are illustrated, respectively. In addition, the timing of the static state determined by the proposed algorithm is presented as well. According to the detection results, the dataset MH_01_easy series is considered to be stationary at 23.5 s, 11.25 s for the MH_03_medium series and 13 s for the MH_04_difficult series. The results also show that the UAVs are not immediately detected as a static state when they are stationary. The reason is that the detection accuracy will significantly affect the subsequent compensation procedure, so the relevant thresholds in the static state detection algorithm are set more severely to guarantee the detection quality.

### 5.2. Motion Compensation Proccess

In order to fit the body frame setup and facilitate the calculation of ${}_{\bar{G}}^{B_{0}}T$, the gravity frame is rotated by −90 degrees according to its *y*-axis first, and the *x*-axis of the rotated gravity frame will be parallel to the opposite direction of gravity. Based on the coordinate configuration, when the UAV is stationary, the relationship between acceleration measurements and gravity can be expressed by

(52) $$\left[\begin{array}{c} a_{aver,x}\\ a_{aver,y}\\ a_{aver,z} \end{array}\right]=R\left[\begin{array}{c} g\\ 0\\ 0 \end{array}\right],$$

where $a_{aver,x}$, $a_{aver,y}$, $a_{aver,z}$ are the average of the latest measurements within 0.5 s in moving window, $\phi_{0}$, $\theta_{0}$ and $\psi_{0}$ are the euler angles of the body frame with respect to the rotated gravity frame in static state, and R is defined as:

(53) $$R=\left[\begin{array}{ccc} \cos\left(\phi_{0}\right) & \sin\left(\phi_{0}\right) & 0\\ -\sin\left(\phi_{0}\right) & \cos\left(\phi_{0}\right) & 0\\ 0 & 0 & 1 \end{array}\right]\left[\begin{array}{ccc} \cos\left(\theta_{0}\right) & 0 & -\sin\left(\theta_{0}\right)\\ 0 & 1 & 0\\ \sin\left(\theta_{0}\right) & 0 & \cos\left(\theta_{0}\right) \end{array}\right]\left[\begin{array}{ccc} 1 & 0 & 0\\ 0 & \cos\left(\psi_{0}\right) & \sin\left(\psi_{0}\right)\\ 0 & -\sin\left(\psi_{0}\right) & \cos\left(\psi_{0}\right) \end{array}\right],$$

Substituting Equation (53) into Equation (52) gives

(54) $$\left[\begin{array}{c} a_{aver,x}\\ a_{aver,y}\\ a_{aver,z} \end{array}\right]=g\left[\begin{array}{c} \cos\left(\phi_{0}\right)\cos\left(\theta_{0}\right)\\ -\sin\left(\phi_{0}\right)\cos\left(\theta_{0}\right)\\ \sin\left(\theta_{0}\right) \end{array}\right],$$

According to Equation (54), $\phi_{0}$ and $\theta_{0}$ can be calculated by

(55) $$\left\{\begin{array}{l} \phi_{0}=\mathrm{atan}2\left(-a_{aver,y},a_{aver,x}\right)\\ \theta_{0}=\mathrm{atan}2\left(a_{aver,z},\sqrt{a_{aver,x}^{2}+a_{aver,y}^{2}}\right) \end{array}\right.,$$

Let $\psi_{0}$ equal to 0 degrees, then the rotation matrix of the first body frame with respect to the gravity frame can be derived as follows

(56) RB0G¯=00−1010100H,

where H is defined as

(57) $$ℋ=\left[\begin{array}{ccc} \cos\left(\theta_{0}\right) & 0 & \sin\left(\theta_{0}\right)\\ 0 & 1 & 0\\ -\sin\left(\theta_{0}\right) & 0 & \cos\left(\theta_{0}\right) \end{array}\right]\left[\begin{array}{ccc} \cos\left(\phi_{0}\right) & -\sin\left(\phi_{0}\right) & 0\\ \sin\left(\phi_{0}\right) & \cos\left(\phi_{0}\right) & 0\\ 0 & 0 & 1 \end{array}\right]$$

Note that the settings of $\psi_{0}$ will not affect the attitude estimation (in Mahony complementary filter). In this paper, we further define that the origin of both the gravity frame and first body frame are coincident, as shown in Equation (58).

(58) $$t_{origin,\bar{G}}={\left[\begin{array}{ccc} 0 & 0 & 0 \end{array}\right]}^{T},$$

According to Equations (56) and (58), TG¯B0 can be obtained as follows

**Figure 16:**
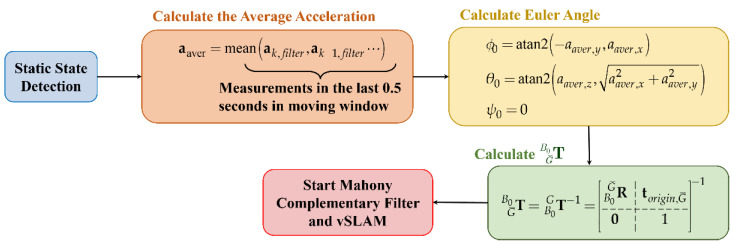
Flowchart of TG¯B0 calculation.

(59) TG¯B0=TB0G¯−1=RB0G¯torigin,G¯01−1,

And the flowchart of the TG¯B0 calculation is illustrated in Figure 16.

The estimated $\phi_{0}$ and $\theta_{0}$ for the EuRoC dataset are listed in Table 5. The errors are calculated by comparing them with the ground truth. According to Table 5, it can be found that the proposed algorithm can accurately detect the stationary state and the associated estimation errors for $\phi_{0}$ and $\theta_{0}$ are almost less than 0.1 degree, which meets the accuracy demand of TG¯B0.

After obtaining the accurate TG¯B0, based on the attitude estimated by Mahony complementary filter, the position information can be derived by integrating the free acceleration twice.

**Figure 17:**
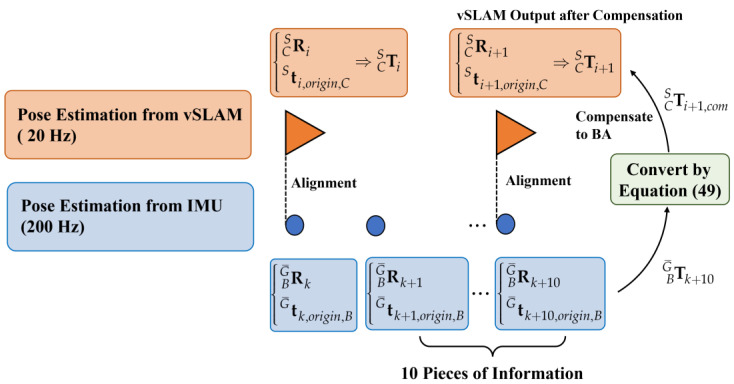
Alignment between vSLAM and IMU data.

(60) tG¯k,origin,B=tG¯k−1,origin,B+vG¯k−1,origin,BΔk+0.5aG¯k−1,freeΔk2vG¯k,origin,B=vG¯k−1,origin,B+aG¯k−1,freeΔk,

where tG¯k,origin,B and vG¯k,origin,B represent the $k^{th}$ position and velocity of the UAV under the gravity frame, respectively, and aG¯k,free is the $k^{th}$ acceleration measurement with gravitational component removed, also known as free acceleration and can be defined as:

(61) aG¯k,free=RBG¯kak,filter−00g,

where RBG¯k is obtained by converting qG¯Bk, which is derived from the Mahony complementary filter. Besides, it is worth mentioning that in the EuRoC dataset, every ten sample periods 10Δk, both IMU and image information will be aligned, which is good timing to fuse them. Therefore, first define TBG¯k+10 as:

(62) TBG¯k+10=RBG¯k+10tG¯k+10,origin,B01,

and convert the reference coordinate system of TBG¯k+10 by Equation (49) to further obtain TCSi+1,com, which will be served as the initial guess of BA in the tracking thread at moment i+1, replacing the constant velocity motion model. These processes are illustrated in Figure 17.

However, due to the double integration used in Equation (60), the position tG¯k,origin,B and velocity vG¯k,origin,B error will accumulate rapidly. Therefore, the position as well as velocity will be reset by the compensated vSLAM output TCSi+1 to reduce the accumulated error. Firstly, convert the reference frame of TCSi+1 by Equation (63).

(63) TBG¯k+10,res=TB0G¯TSB0TCSi+1TBC,

where TBG¯k+10,res is defined as:

(64) TBG¯k+10,res=RBG¯k+10,restG¯k+10,res01,

Thus, the reset position tG¯k+10,res can be obtained, while the reset speed vG¯k+10,res can be derived from the previous reset position, as shown in Equation (65).

**Figure 18:**
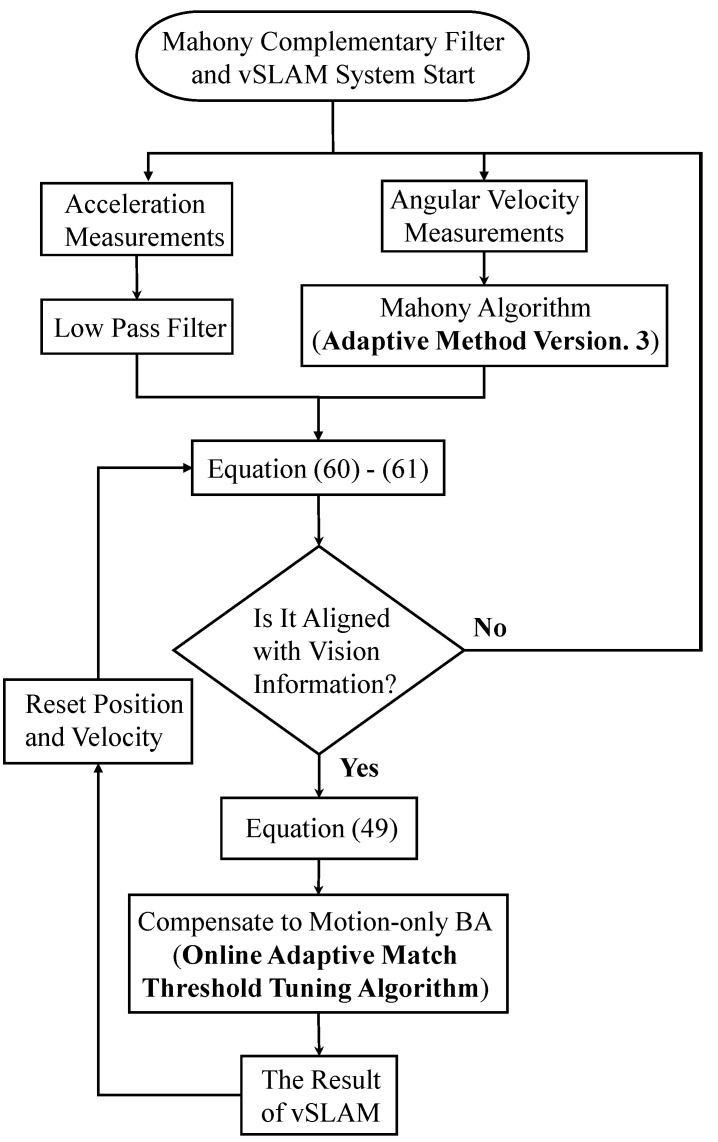
Real-time motion compensation loop subroutine.

(65) vG¯k+10,res=tG¯k+10,res−tG¯k,res10Δk,

After obtaining the reset position and reset speed, Equation (60) can be performed again based on them. The overall compensation subroutine working in real-time is illustrated in Figure 18.

## 6. Experiment Verification

In this section, the advantages of the proposed algorithm are verified by using the EuRoC dataset. Not only the pose estimation accuracy, FPS and anti-shading robustness are examined, but also the online adaptive tuning process of both matching thresholds and $K_{p}$ is shown to give the readers a more comprehensive understanding of the proposed algorithm.

### 6.1. Ablation Studied for Accuracy Comparison

This section mainly analyzes the estimation accuracy of the following four different strategies so as to independently evaluate the effort of both online adaptive tuning algorithms:

- Case. 1: not to use both proposed online adaptive tuning algorithms.
- Case. 2: only use the online adaptive matching threshold tuning algorithm.
- Case. 3: only use the online adaptive $K_{p}$ tuning algorithm.
- Case. 4: use both proposed online adaptive tuning algorithms.

**Figure 19:**
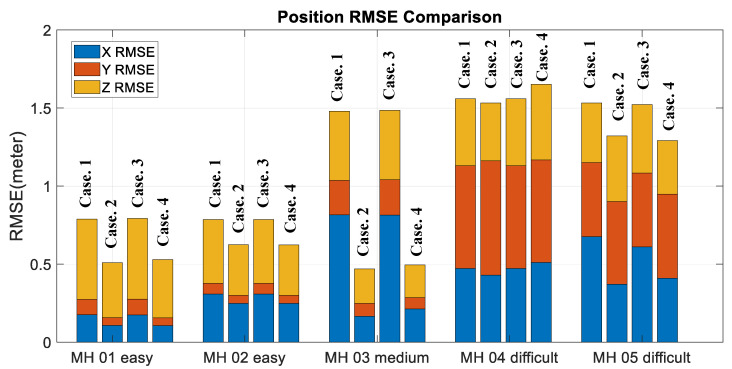
Position accuracy comparison.

**Figure 20:**
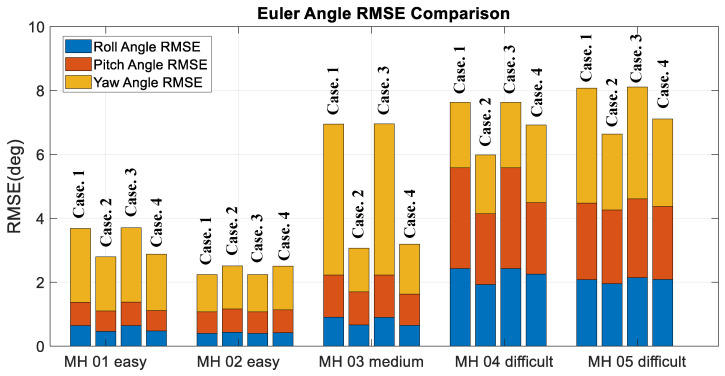
Euler angle accuracy comparison.

**Figure 21:**
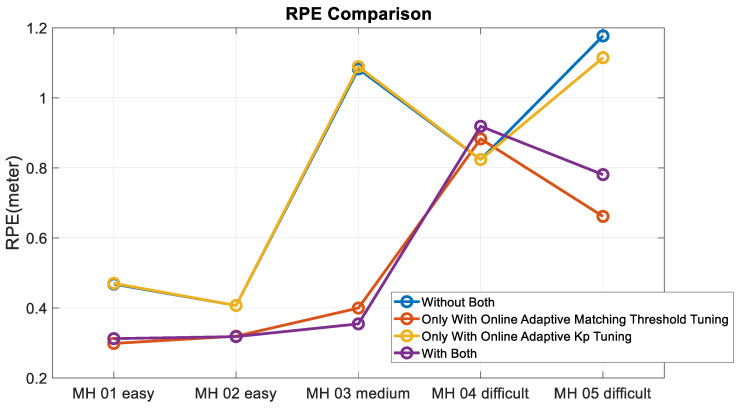
RPE comparison.

**Figure 22:**
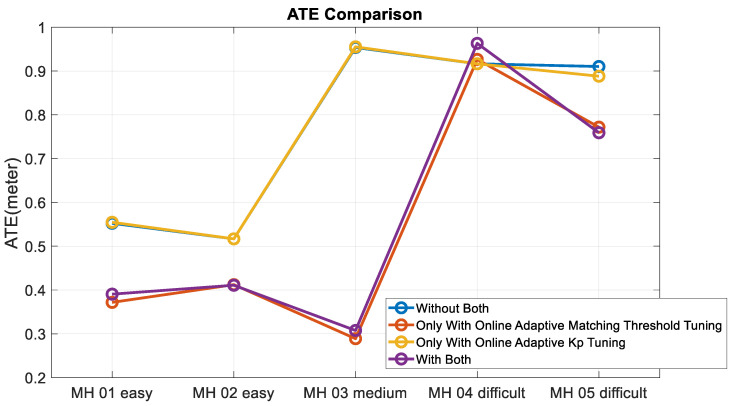
ATE comparison.

Moreover, to validate the contribution of the proposed algorithm for localization accuracy objectively, the loop closure for drift compensation in the following experiments is not considered. Based on the above four conditions, the corresponding accuracy analysis results are shown in Figure 19, Figure 20, Figure 21 and Figure 22. In Figure 19 and Figure 20, the error bars in each dataset series with respect to the above four different strategies are displayed from left to right.

**Figure 23:**
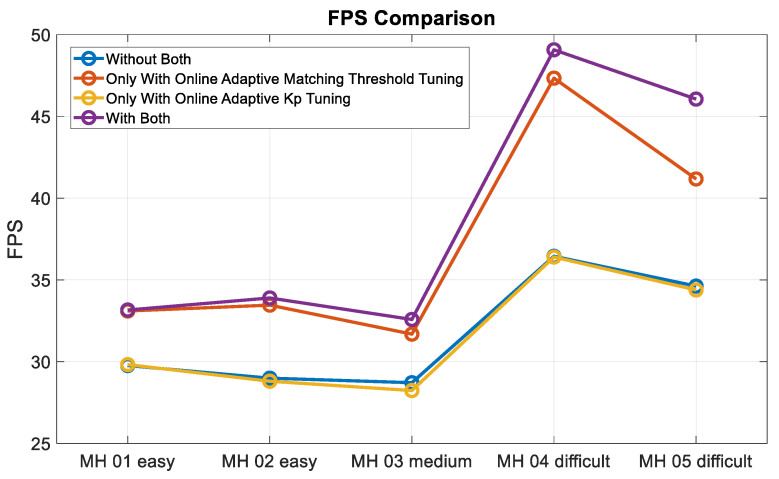
FPS comparison.

Experimental examinations clearly show that the proposed online adaptive matching threshold tuning algorithm can effectively improve the accuracy. In particular, in the MH_03_medium series, 63% and 70% accuracy improvement is achieved in RPE and ATE, respectively. Even though the accuracy did not improve in the MH_04_difficult series, the overall accuracy did not change drastically too much, and meanwhile, according to Figure 20, the attitude estimation accuracy is still improved. However, we also know that the online adaptive $K_{p}$ tuning algorithm is not significantly helpful for accuracy improvement. Even though in Table 4, the IMU data in each dataset series has been tested independently, and results show that the online adaptive $K_{p}$ tuning algorithm can improve the attitude estimation accuracy effectively. Based on the conclusion given in Figure 9, it can be further inferred that since the motion-only BA with Huber robust kernel significantly reduces the sensitivity to the initial value, the pose compensation with IMU does not have a noticeable effect. However, for severe situations, such as when the stereo cameras are temporarily occluded, the IMU’s pose compensation will play an important role in maintaining the positioning stability, which will be tested and analyzed in the next section. Figure 23 shows the time cost for the four different strategies, while Table 6 lists the computer specifications used in this paper. Besides, from the onboard implementation point of view, the Intel NUC, a lightweight and small computer with comparable computing power, is sufficient to carry out the proposed algorithms. Figure 23 clearly shows that the online adaptive matching threshold tuning algorithm can improve FPS effectively. The reason is that the proposed adaptive matching threshold tuning algorithm can reasonably adjust the matching threshold to avoid over-triggering the keyframe selection, namely excessive mapping and involving too many map points that need optimization.

**Figure 24:**
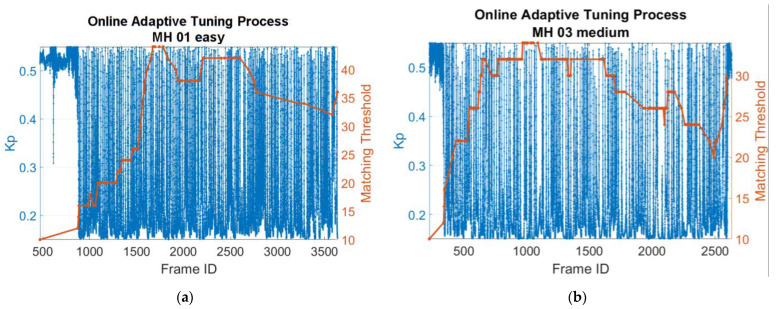
Adaptive tuning process of matching threshold and $K_{p}$: (**a**) For MH_01_easy series; (**b**) For MH_03_medium series.

Figure 24 shows the automatic tuning process of matching threshold and $K_{p}$ parameter (take the MH_01_easy and MH_03_medium series, for example). Figure 24 shows the matching threshold is not always being adjusted at every frame, but only when the keyframe is established, which corresponds to the content described in Section 3.2. In contrast, the online adaptive $K_{p}$ tuning is performed at every moment, as long as the acceleration information is available.

### 6.2. Anti-Shading Robustness Test

Since the IMU information is integrated into the vSLAM algorithm, the UAVs’ flight trajectories can temporarily rely on the motion compensation loop, which further enhances the overall anti-shading robustness of the original vSLAM. Following the above statement, the robustness of the overall system against shading will be examined. The examination method is to turn off the left and right camera images simultaneously to simulate the shading situation. In order to analyze the anti-shading performance more comprehensively, the following three vSLAM scenarios will be examined and analyzed.

- Case. A: without using both the online adaptive parameter tuning algorithm and the motion compensation loop subroutine.
- Case. B: only using motion compensation loop subroutine.
- Case. C: using the proposed online adaptive parameter tuning algorithm and the motion compensation loop subroutine.

Table 7 shows the corresponding results, including the time stamp for image loss, and the pose accuracy represented in RPE and ATE.

According to Table 7, Case. A, the original vSLAM system, is unable to perform localization successfully without using both the proposed online adaptive algorithm and the motion compensation loop subroutine. In addition, in Case B, even though the system can work properly in MH_01_easy, MH_02_easy and MH_05_difficult series, the UAVs’ localizations still fail for MH_03_medium and MH_04_difficult series. On the contrary, by using the proposed online adaptive algorithm and the motion compensation loop subroutine, namely Case. C, not only the pose estimation can be successfully conducted in all data series, but also the localization accuracy can be maintained. The above experimental results firmly reveal that the developed motion compensation loop subroutine can indeed increase the robustness against shading by the addition of IMU information, and more importantly, a better level of robustness performance will be achieved by further combining online adaptive parameter tuning algorithms. The key factor is that the online adaptive parameter tuning algorithm can effectively increase the attitude estimation accuracy, thereby improving the motion compensation quality, which heavily relies on the estimated free acceleration and attitude shown in Equation (61).

**Figure 25:**
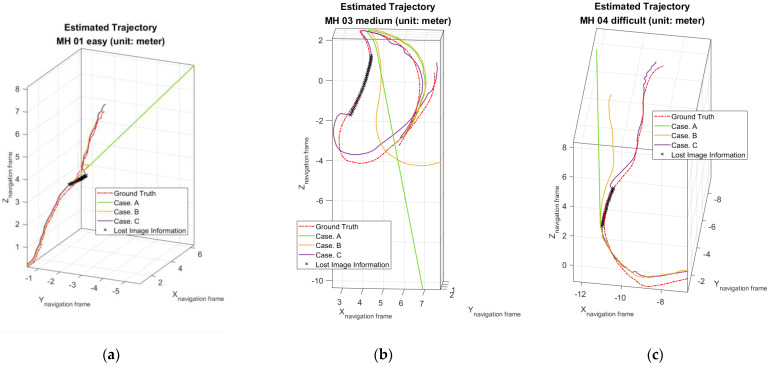
Estimated trajectories in anti−shading robustness test: (**a**) For MH_01_easy series; (**b**) For MH_03_medium series; (**c**) For MH_04_difficult series.

The trajectories of the anti-shading experiment are shown in Figure 25. The black crosses in these figures represent the localization results (Case. C) via using the proposed algorithm during the critical image loss period. These figures also show that the positioning system without the proposed algorithm not only fails to track after image loss, but also makes a straight-line estimation based on the constant velocity motion model. This experiment implies that there will be less chance to pull back the trajectory under aggressive motions even when the image information recovers. On the contrary, these failures can be solved successfully by applying the proposed method.

**Figure 26:**
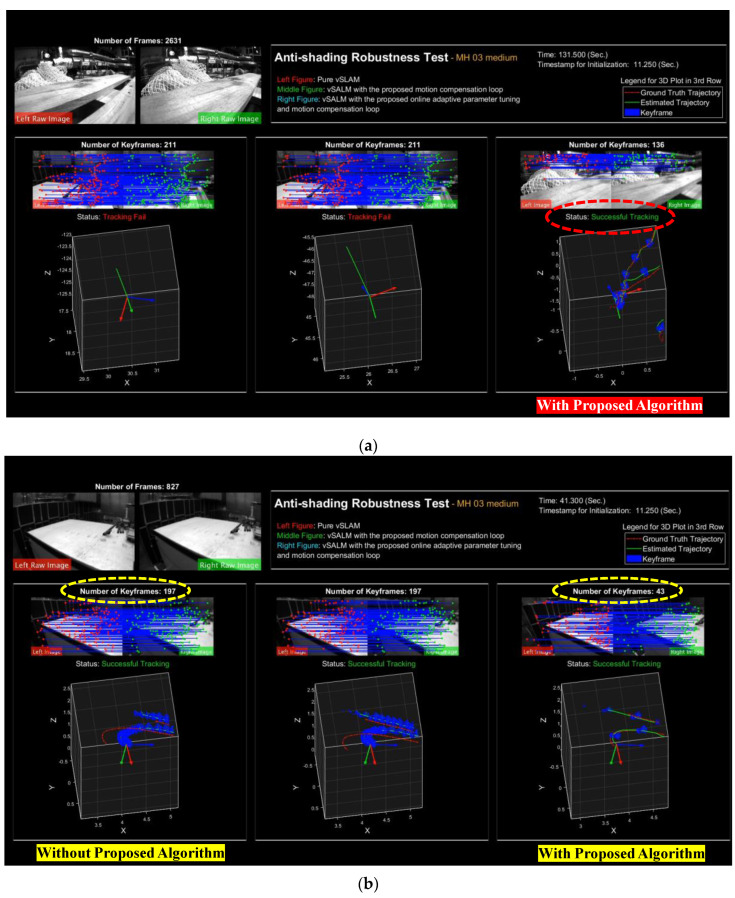
The results of the anti-shading robustness test with MH_03_medium series: (**a**) Anti-shading robustness examination (Supplementary File Vedio S1); (**b**) Key frame examination (*X*-*Y*-*Z* axis Unit: meter).

In the following, we present the results of anti-shading robustness tests in parallel, as shown in Figure 26, to make the robustness analysis of the proposed vSLAM more intuitive. Figure 26a shows the localization results for three different scenarios after masking the stereo camera at the 849th frame in the MH_03_medium series. It is obvious that the proposed algorithm is the only survivor that completes the indoor flight localization and mapping examination successfully, while the others all fail to track the flight trajectories; this result highlights again that the proposed algorithm is effective in improving the robustness against shading for the vSLAM system. In addition, Figure 26b shows the localization results for three different scenarios before the stereo camera is masked. Even though all these three scenarios conduct localization successfully, the proposed algorithm has the lowest number of keyframes, which implies that the developed vSLAM algorithm can preserve precise vSLAM with fewer map feature points. It is worth mentioning that fewer map feature points requirement reduces the computational loading and saves a lot of storage memory space; these advantages make the proposed algorithm more suitable for practical applications of light UAVs.

## 7. Conclusions

UAVs are one of the most promising vehicles in recent years, and the underlying positioning technology is receiving more and more attention. However, the over-reliance on GPS has limited the application diversity of UAVs. In order to solve the navigation problem in GPS-denied or interferenced environments, the stereo vSLAM solution is gradually gaining attention. Therefore, this paper presents an improved UAVs vSLAM system based on the S-PTAM architecture and Mahony complementary filter. The proposed online adaptive matching threshold tuning algorithm and online adaptive $K_{p}$ tuning algorithm can improve the overall vSLAM accuracy and robustness. These adaptive tuning algorithms will automatically adjust the influential matching threshold online and the $K_{p}$ gain used in the Mahony complementary filter, respectively. The adaptive mechanisms eliminate the tedious manual adjustment of these non-physically meaningful parameters. In order to fuse the IMU and vSLAM information, an additional static state detection algorithm is proposed, which has been tested and proven to be accurate in detecting static state. After the static state has been detected, an accurate initial relationship can be computed, which will greatly help the subsequent information conversion. Besides, the error of the initial relationship is almost less 0.1 degree. Based on this accurate initial relationship, a good conversion mechanism between IMU and vSLAM is established. Finally, a couple of experimental studies are used to validate the proposed algorithm and the vSLAM architecture. The results show that the online adaptive matching threshold tuning algorithm can improve localization accuracy and FPS effectively. Moreover, an anti-shading robustness test was further addressed. Experiments firmly show that the vSLAM robustness against temporary image loss can be achieved successfully by incorporating the proposed algorithms.

## Figures and Tables

**Table 1:** Accuracy comparison according to the different matching thresholds for MH_01_easy series.

Series	Error Type	Matching Threshold=10	Matching Threshold=15	Matching Threshold=20
MH_01_easy	RPE	0.483251	0.503435	0.404268
	ATE	0.578836	0.615321	0.503354

**Table 2:** Parameter settings for the proposed online adaptive matching threshold tuning algorithm.

Parameter	Setting Value
$thres_{m,1}$	0.65
$thres_{yaw,1}$	5
$thres_{m,2}$	1
$thres_{yaw,2}$	6
$thres_{max}$	45
$thres_{min}$	5

**Table 3:** Parameter setting for the proposed online adaptive $K_{p}$ tuning algorithm.

Parameter	Setting Value
$thres_{norm}$	0.01
$K_{p}$	0.15
κ	12
$\Delta K_{p}$ *	0.4

* In MH_02_easy and MH_03_medium series, it will be set to 0.01.

**Table 4:** Results of online adaptive $K_{p}$ tuning algorithm for EuRoC dataset. (Unit: deg).

Method\Series	MH_01_Easy		MH_02_Easy		MH_03_Medium		MH_04_Difficult		MH_05_Difficult	
	Roll (RMSE)	Pitch (RMSE)	Roll (RMSE)	Pitch (RMSE)	Roll (RMSE)	Pitch (RMSE)	Roll (RMSE)	Pitch (RMSE)	Roll (RMSE)	Pitch (RMSE)
Pure Integration	1.2140	0.8997	0.3969	0.3090	0.1739	0.2019	0.28612	0.2080	0.32292	0.2605
Arctan Method	2.0094	1.7620	1.5191	1.6333	4.3881	3.9834	2.4454	2.6123	2.4323	2.4165
Pure Mahony	0.35373	0.2963	0.2513	0.3500	0.88704	0.6433	0.72719	0.5796	0.6052	0.5560
Conditional Method	1.1734	0.5215	0.3013	0.2087	0.1246	0.1592	0.2944	0.1838	0.2704	0.2456
Adaptive Method Version.1	1.1634	0.5930	0.3226	0.2182	0.1272	0.1651	0.2949	0.1899	0.2847	0.2471
Adaptive Method Version. 2	1.1724	0.5270	0.30324	0.2091	0.12412	0.1595	0.2946	0.1845	0.2718	0.2457
Adaptive Method Version. 3	1.0487	0.4166	0.29874	0.2084	0.12569	0.1590	0.2928	0.1802	0.2216	0.2448

**Table 5:** Results of a static state detection algorithm for the EuRoC dataset.

Series	Time Stamp (Second) for Triggering Static State Detection Algorithm (Checked from Figure 15)	The True State of The UAV is Stationary or Not (Checked from Images)	$\mathrm{Estimated}\;\phi_{0}$ $\;\mathrm{and}\;\theta_{0}\;(\deg)$		Angle Error (deg) *
MH_01_easy	23.5	yes	$\phi_{0}$	4.0044	−0.008034
			$\theta_{0}$	15.6614	−0.034309
MH_02_easy	28.75	yes	$\phi_{0}$	3.3513	0.001947
			$\theta_{0}$	15.5490	−0.047756
MH_03_medium	11.25	yes	$\phi_{0}$	4.8658	0.050396
			$\theta_{0}$	15.6173	−0.045054
MH_04_difficult	13	yes	$\phi_{0}$	0.4097	−0.073378
			$\theta_{0}$	24.1770	0.113550
MH_05_ difficult	14.75	yes	$\phi_{0}$	0.1934	−0.006640
			$\theta_{0}$	23.9930	0.095132

* The error is defined as the ground truth minus the estimated value.

**Table 6:** Computer specifications used for the proposed algorithm.

CPU	**RAM**
Intel Core i7-11800H @2.30GHz	16 GB

**Table 7:** Results of anti-shading robustness test.

Scenarios	Series	Time Stamp (Second) for Image Loss	RPE (Meter)	ATE (Meter)
Case. A (Pure vSLAM)	MH_01_easy	55.45~56.95	Tracking Fail	Tracking Fail
	MH_02_easy	55.45~57.95	Tracking Fail	Tracking Fail
	MH_03_medium	42.35~44.35	Tracking Fail	Tracking Fail
	MH_04_difficult	34.95~36.45	Tracking Fail	Tracking Fail
	MH_05_ difficult	74.95~77.45	Tracking Fail	Tracking Fail
Case. B (vSALM with the proposed motion compensation loop)	MH_01_easy	55.45~56.95	0.5200	0.6119
	MH_02_easy	55.45~57.95	0.4210	0.5292
	MH_03_medium	42.35~44.35	Tracking Fail	Tracking Fail
	MH_04_difficult	34.95~36.45	Tracking Fail	Tracking Fail
	MH_05_ difficult	74.95~77.45	1.1378	**0.8813**
Case. C (vSALM with the proposed online adaptive parameter tuning and motion compensation loop)	MH_01_easy	55.45~56.95	0.2578	0.3290
	MH_02_easy	55.45~57.95	0.3686	0.447 1
	MH_03_medium	42.35~44.35	0.424 8	0.4615
	MH_04_difficult	34.95~36.45	0.7099	0.779 4
	MH_05_ difficult	74.95~77.45	1.0806	0.8940

## Data Availability

The official website of the EuRoC dataset, including downloadable links is https://projects.asl.ethz.ch/datasets/doku.php?id=kmavvisualinertialdatasets (accessed on 10 October 2022); EuRoC dataset provides stereo image sequences, IMU measurements, ground truth about 6 DoF pose and relative pose relationships between sensors which are included in the sensor.yaml file in each dataset series folder; The information about sensor type, UAVs used, dataset series characteristics and other details can be accessed in [27] or the official website presented above.

## Supplementary Materials

The following supporting information can be downloaded at: https://www.mdpi.com/article/10.3390/s22208067/s1, Vedio S1: An Online Adaptive Parameter Tuning vSLAM Algorithm for UAVs in GPS-denied Environments-Series (3).
